# Role of Lipids in Morphogenesis of T-Cell Microvilli

**DOI:** 10.3389/fimmu.2021.613591

**Published:** 2021-03-10

**Authors:** Marek Cebecauer

**Affiliations:** Department of Biophysical Chemistry, J. Heyrovsky Institute of Physical Chemistry of the Czech Academy of Sciences (CAS), Prague, Czechia

**Keywords:** T cell, microvilli, sphingolipids, phosphoinositides, lipid rafts, membrane curvature, dimpling domains, membrane-associated proteins

## Abstract

T cells communicate with the environment *via* surface receptors. Cooperation of surface receptors regulates T-cell responses to diverse stimuli. Recently, finger-like membrane protrusions, microvilli, have been demonstrated to play a role in the organization of receptors and, hence, T-cell activation. However, little is known about the morphogenesis of dynamic microvilli, especially in the cells of immune system. In this review, I focus on the potential role of lipids and lipid domains in morphogenesis of microvilli. Discussed is the option that clustering of sphingolipids with phosphoinositides at the plasma membrane results in dimpling (curved) domains. Such domains can attract phosphoinositide-binding proteins and stimulate actin cytoskeleton reorganization. This process triggers cortical actin opening and bundling of actin fibres to support the growing of microvilli. Critical regulators of microvilli morphogenesis in T cells are unknown. At the end, I suggest several candidates with a potential to organize proteins and lipids in these structures.

## Introduction

T lymphocytes, important supervisors of the immune system, are activated and regulated through receptors expressed on their surface. Surface of lymphocytes is densely covered by membrane protrusions, mainly microvilli (1, 2), which allow for a more complex three-dimensional (3D) organization of receptors compared to a flat membrane. Indeed, critical receptors of T-cell activation, T cell receptor (TCR), CD2, CD4 and CD28 were shown to accumulate at the tips of microvilli in recent studies benefitting from 3D imaging at high resolution (1, 3–7). On the contrary, CD45 is excluded from these areas (6, 7). It was suggested that non-random 3D distribution of receptors is important for optimisation of signalling and cellular responses (8–10). However, little is known about the origin of microvilli and molecules involved in their formation and homeostasis in T lymphocytes. Insight into molecular biophysics and structural details of these membrane protrusions can help to better understand T-cell function in health and disease.

In this work, I suggest the role of lipids and lipid domains in deformation of membranes and their potential role in the formation and organization of microvilli. I start with a brief introduction to microvilli structure and function. These data almost exclusively originate from studies of microvilli in epithelial cells. It is thus important to note here that microvilli of epithelial cells are more stable and may differ in structural details when compared to microvilli on leukocytes. In the central sections, I hypothezise a role of curved lipid domains in microvilli formation and describe regulatory role of lipids for the function of proteins localized prevalently to these structures. I finish by discussing a handful of molecules with a potential role in morphogenesis of T-cell microvilli. Like lipid domains, function of these proteins in T cells needs to be determined.

## Microvilli and Their Structure

**Figure 1:**
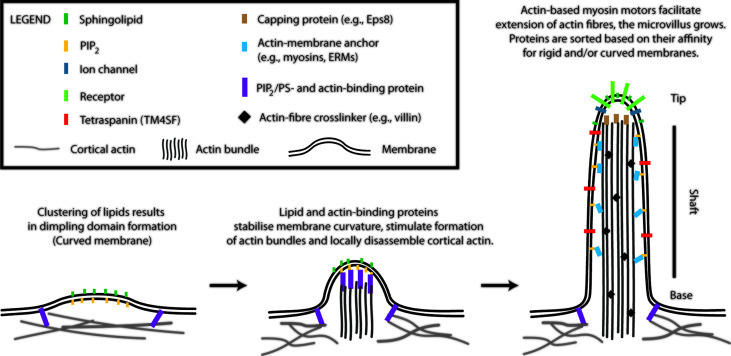
A model of T-cell microvilli morphogenesis. The onset of microvilli can be triggered by transient formation of dimpling lipid domains (see also **Figure 2**). Sphingolipids together with phosphoinositides (e.g., phosphatidylinositol 4,5-bisphosphate; PIP_2_) own a high potential to form dimpling domains in asymmetric membranes. Later, actin-binding proteins, which associate with plasma membrane *via* PIP_2_ (or phosphatidylserine), induce cortical actin opening and stabilize dimpling domains. Similar proteins can stimulate bundling of actin fibres. The growth of microvilli is driven by polymerization of actin at the plus end of the fibres (distal end of microvilli). Myosins dynamically anchor actin bundles to the membrane at the shaft. ERM (ezrin, radixin, and moesin) proteins function in a similar fashion (membrane anchor) and regulate stability of microvilli. Proteins with affinity for rigid (sphingolipid-enriched) and/or for curved membranes accumulate at the tip or shaft of microvilli. Little is known about T-cell signalling molecules in the lumen of microvilli. The existence of terminal web in microvilli of leukocytes remains unknown. Components of microvilli are not drawn in scale.

Microvilli are finger-like membrane protrusions at the surface of metazoan cells (11). Microvilli consist of the tip, shaft, and base, which connects these structures to the plasma membrane and cortical actin (**Figure 1**). Actin bundles determine a shape of microvilli and are responsible for their stability, but also a dynamic character. In the shaft, the membrane is tightly linked to actin bundles *via* actin- and membrane-binding proteins [e.g., myosins and ERM proteins; see **Figure 1** (12)]. At the base, at least in epithelial cells, actin bundle terminates in the network of intermediate filaments known as ‘terminal web’ (13, 14). The size of microvilli is regulated by the growth of actin fibres at the tip (15). Microvilli on the surface of polarized epithelial cells covering organs in direct contact with the exterior are rather stable and long (11). Microvilli on T cells are smaller and highly dynamic (1, 2, 16, 17). With ~100 nm in a diameter and a length of 0.5–5 µm, they represent rather small surface structures [Figure 1 in (17) and Figure 1 in (1)]. It is their abundance and flexibility, which makes these structures important for T-cell function. For example, vesicles with receptors and other effector molecules can be shed off the microvilli tips. This phenomenon was observed in epithelia, as well as in T cells, and can be part of complex regulatory mechanisms in multicellular organisms (1, 18).

The accumulation of receptors at the tip of microvilli is beneficiary for an easy access to ligands, substrates or mechanical forces and can determine cellular responses to such stimuli (8, 9, 11). The shaft and the base potentially function as a selecting region, segregating molecules to different membrane environments. However, physico-chemical basis of such selection remains unknown. Importantly, it is still unclear what defines a local onset and chemical composition of microvilli. In the following sections, I suggest a model (**Figure 1**), in which membrane lipids and their physico-chemical properties trigger the onset of microvilli formation.

## Lipid Domains and Local Bending of Membranes

**Figure 2:**
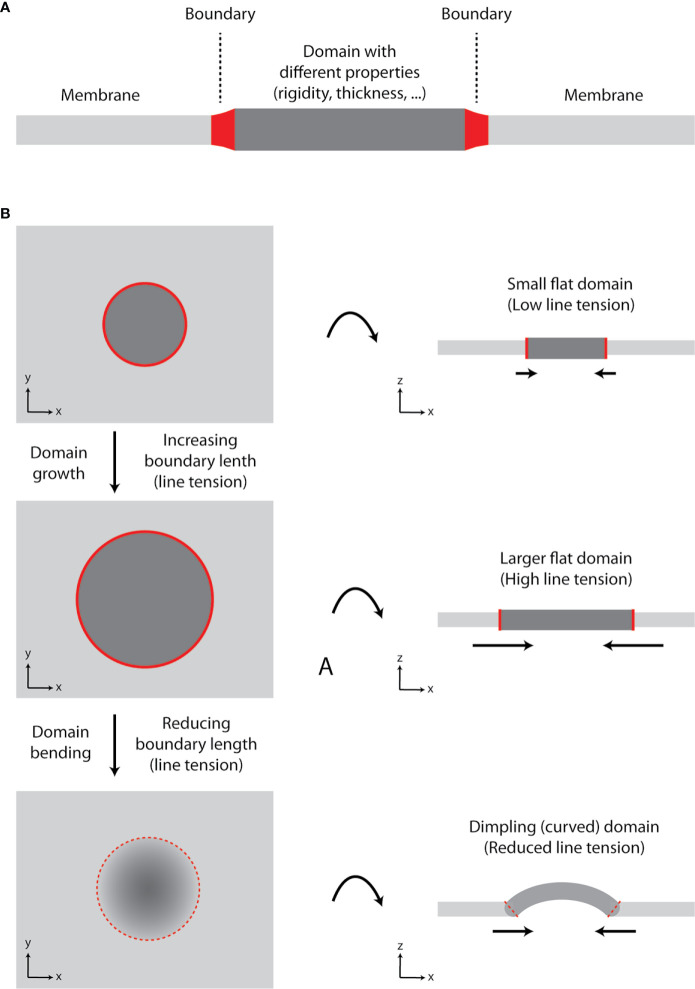
Growth of lipid nanodomains and dimpling (curved) domain formation. **(A)** Schematic illustration of lipid membrane with a domain. The domain has different physico-chemical properties (e.g., rigidity-conformational order, thickness) compared to the surrounding lipid bilayer. The two environments are separated by the boundary. **(B)** Schematic illustration of a domain growth and formation of a dimpling domain. Certain lipids (e.g., sphingolipids and cholesterol) tend to segregate into circular domains in synthetic membranes containing unsaturated glycerophospholipids due to their immiscibility at lower temperatures or in the presence of other clustering factors (e.g., proteins). As the domain grows, line tension at the boundary increases, until it reaches the point, at which it exceeds bending energy required for membrane deformation and dimpling domain is formed. The length of the boundary is reduced, and further growth of the membrane is accompanied by membrane tubulation, but not increase in line tension. Hence, domain formation can lead to induction of membrane curvature and its tubulation.

In our review on membrane lipid nanodomains [(19), Section 8.4], we discussed a role of curvature in stabilization of domains and prevention of their fusion. In general, formation of a lipid domain with different properties (e.g., rigidity and thickness) compared to the adjacent membrane results in line tension at the boundary (borderline) between the two ‘phases’ (**Figure 2A**). In a growing domain, the length of boundary increases, and line tension rises. However, lipid membranes prefer to minimize tensions associated with their organization (20, 21). Since elastic properties (bending modulus) of membranes are not changing significantly, the size of a domain can reach the point, at which membrane starts to bend and form dimpling domains [**Figure 2B** (22, 23)]. This is caused by the fact that line tension at the boundary exceeds the bending energy (resistance) of a membrane required for its deformation. Membrane bending reduces the boundary length and, thus, line tension. Further growth of a domain is enabled by enhanced curvature, which can result in membrane tubulation. The length of boundary and line tension remain constant for such growing domain/protrusion (21).

In flat membranes, small domains diminish due to their fusion into larger entities, as observed in model, phase-separated giant unilamellar vesicles (24). Fusion of small domains reduces the length of boundary and line tension (23). However, large lipid domains are not frequent in cells. It is currently agreed that the plasma membrane is highly heterogenous due to the presence of small (nanometric) domains (25, 26). One can thus speculate that plasma membrane is prone to form dimpling domains, which cannot fuse due to repulsive forces at their boundaries (19, 22).

## Clustering of Sphingolipids and Phosphoinositides Triggers Microvilli Formation

Cellular membranes are composed of a large variety of lipid species. Among those, sphingolipids, with their long and saturated acyl chains and affinity to cholesterol, are prone to segregate from unsaturated glycerophospholipids and form nanodomains (19, 27–29). Ikenouchi and colleagues suggested that sphingolipids are required for the existence of microvilli and, potentially, also initiation of their formation in epithelial cells (30). Conversion of sphingomyelin to ceramides by acidic sphingomyelinase in these cells led to impaired microvilli. In untreated cells, sphingolipids accumulated on microvilli (30). Accumulation of sphingomyelin (and cholesterol) in microvilli was confirmed in another study, which employed lysenin labeling of sphingomyelin (perfolysin *O* for cholesterol) and sensitive nanoSIMS imaging in CHO-K1 epithelial-like cells (31). Of note, only freely accessible lipids could be detected using this method. In another study, interference with sphingolipid or cholesterol synthesis lead to reduced presence of microvilli on epithelial cells (32). All these studies indicate that sphingolipids are essential for the morphogenesis of microvilli.

Membrane lipid composition considerably differs between various cell types. Though sphingolipids consistently constitute 20-40% of plasma membrane lipids (33, 34). Local concentration of sphingolipids is even higher due to chemical asymmetry of the plasma membrane (lipid bilayer). Such high content of sphingolipids in the outer leaflet can lead to their transient clustering and, occasionally, formation of dimpling domains. Indeed, bilayer asymmetry reduces bending modulus of a membrane and, thus, facilitates its deformation (22, 35). In cells, phosphatidylinositol 4,5-bisphosphate (PIP_2_) molecules were found to cluster underneath sphingolipid domains during membrane deformation induced by viral proteins [virion budding (36, 37)]. PIP_2_ was also found to accumulate in microvilli (30). But comprehensive analysis of lipids in microvilli has not been performed to date (38). Therefore, it is unclear what is the content of PIP_2_, sphingolipids and other lipids (e.g., cholesterol) in these structures.

The presence of PIP_2_ in the apical membrane of epithelial cells, but of phosphatidylinositol 3,4,5-trisphosphate (PIP_3_) in basolateral membrane, further supports the involvement of this lipid in microvilli formation (39). Microvilli can be found only on the apical surface of epithelial cells. Apical membrane of polarized cells is also enriched in sphingomyelin and cholesterol (40). Moreover, PIP_2_ accumulates in the uropod of motile cells, whereas PIP_3_ can be found in the leading edge. Microvilli are often observed at the back of motile cells, including T cells (17, 41, 42). In analogy to sphingomyelin domains, cholesterol facilitates clustering of PIP_2_ (43). Due to its high lateral and transbilayer mobility (44, 45), cholesterol is expected to freely access dimpling domains. Interestingly, cholesterol does not influence bending modulus of synthetic membranes with diverse lipid composition (46, 47). Thus, cholesterol does not directly raise the energy required for membrane deformation and establishment of dimpling membranes, but the effect can depend on its intramembrane orientation and distribution between the outer and inner leaflet (45).

## Lipid-Protein Crosstalk in Microvillar Morphogenesis and Function

To further highlight the importance of lipids in microvilli morphogenesis, I will describe three examples where lipid metabolism determines the function of critical proteins in microvilli. The examples were selected based on the depth of our understanding of these regulatory processes. As in the case of microvilli structure, this knowledge comes from microvilli of epithelial cells, but similar regulatory mechanisms can be expected in T-cells.

ERM family proteins (ezrin, radixin, and moesin) tightly anchor actin-bundles to the membrane of microvilli. This is facilitated by binding of their FERM domain to PIP_2_ (48). The process is regulated by a local lipid environment. Conversion of sphingomyelin to ceramide and of sphingosine to sphingosine-1-phosphate negatively and positively, respectively, regulate membrane-association of ERM proteins and, thus, stability of microvilli (49, 50). The role of ERM proteins for microvilli is evidently critical, since their knock-down leads to their reduced size and number (51, 52).

Podocalyxin-1 accumulates in microvilli of epithelial cells. Podocalyxin-1 interacts with ERM proteins *via* EBP50 (53). It further interacts with phosphoinositide-4-phosphate 5-kinase (PI5K) β and delivers this critical enzyme to microvilli. The formation of podocalyxin-1 multiprotein complex with PI5K leads to a local increase in PIP_2_ synthesis and stability of microvilli (30). Interestingly, podocalyxin-1 associates with sphingolipid domains, probably upon its palmitoylation (54). The crosstalk of diverse lipids in the regulation of this protein remains unknown.

Another protein associating with sphingolipid domains on microvilli is prominin-1 [also called CD133 (55)]. Overexpression of prominin-1 increases a number of microvilli (56). This protein directly binds cholesterol and GM1 ganglioside (57). These lipid-protein interactions were found essential for fine tuning of microvillar structure. The protein is further regulated by phosphorylation of its regulatory tyrosines [Y_817_/Y_828_ (56)]. Phosphorylation of these tyrosines regulates interaction of prominin-1 with phosphoinositide 3-kinase (PI3K). In contrast to PI5K, PI3K locally reduces available PIP_2_ by its conversion to PIP_3_ and destabilizes the anchorage of actin bundles to the membrane (56).

## Potential Regulators of Microvilli in T Cells

I have argued above that lipid domains induce curvature in flat regions of the plasma membrane. Such domains would be transient in the absence of supporting proteins (**Figure 1**). The process is well described for the endocytosis or viral budding (36, 37, 58–61). For example, matrix proteins (e.g., Gag of HIV-1) form a dome-like structure under the curved membrane of nascent viral particles.

Proteins stabilizing dimpling domains at the sites of newly assembling microvilli have not been described yet. A few proteins (e.g., prominin-1/CD133, podocalyxin-1) reported to regulate microvilli morphogenesis in epithelial cells (30, 55), are not expressed in T cells or at highly variable levels in diverse T-cell subsets. Their role in microvilli morphogenesis in T cells is thus questionable. Here, I will focus on four proteins (protein families), which exhibit great potential to induce or stabilize microvilli in T cells.

The geometry and chemistry of dimpling domains delineates properties of potential supporting proteins. These must interact with negative curvature and anionic lipids. I-BAR domain proteins exhibit such properties. IRSp53 contains I-BAR domain and was shown to induce negative curvature and tubulation in synthetic vesicles (62). IRSp53 localizes to curved membranes of neuronal cells (63) and filopodia of motile fibroblasts (64). It supports membrane ruffling and protrusions in T cells (65). In epithelial cells, it is expressed at the microvilli-containing apical membrane and functionally associates with podocalyxin-1 (66). As a protein of countless functions, it will be important to characterize its specific role in microvilli of T cells. Alternatively, other I-BAR domain-containing proteins can fulfil this function in lymphoid cells.

Tetherin (also called CD317) with affinity for ordered, sphingolipid-rich membranes interacts with BAR domain-containing RICH family proteins (67). Tetherin/RICH-2 complex forms a mechanical support of epithelial microvilli (68). Its analog, RICH-1, is expressed in T-cells (Human Protein Atlas). BAR domain of RICH proteins can induce positive curvature and tubulate lipid vesicles containing PIP_2_ in the absence of tetherin (69). The potential of tetherin/RICH complex thus lies at the neck connecting microvilli (or dimpling domains) to membrane base *via* a positively curved segment (**Figure 1**).

Unconventional myosins (e.g., myo1a, myo7b) link actin fibres to membrane by their interaction with anionic lipids, PIP_2_ or phosphatidylserine (70). Myosins also contribute to the formation of a ‘hole’ in the cortical actin at the site of new microvillus formation (71). Such local depletion of cortical actin is essential for the initiation of membrane protrusions (72). This process may be also connected to the formation and stabilization of dimpling lipid domains.

Members of tetraspanin protein superfamily (TM4SF) accumulate at the microvilli of diverse cells. CD9, CD81, CD82, and TSPAN33 were shown to control the size and shape of microvilli in both, leukocytes and epithelial cells (73–75). TM4SF proteins (e.g., CD81) require highly curved membrane for their assembly into virus-like particles induced by HIV-1 Gag protein (37, 76). The main role of TM4SF is thus expected for growing or established microvilli with highly curved tubular membrane.

None of the proteins mentioned in this section was already determined as a microvilli regulator in T cells. However, I believe that intense research in this direction may soon offer interesting discoveries related not only to microvilli, but also to T-cell signaling and function.

## Conclusions

Recent observations demonstrate that microvilli play essential role in T-cell activation. Key signalling molecules were found to accumulate in different parts of these morphological structures. Theoretical and biophysical studies indicate that sphingolipids and phosphoinositides in complex asymmetric membranes tend to generate dimpling domains. In the plasma membrane of T cells, dimpling domains can be the sites of an onset of microvilli, as indicated in the presented model. Specific lipids also fine tune behaviour of critical regulatory proteins in microvilli. These data substantiate the role of lipids in morphogenesis and function of microvilli. However, in T cells, the identity of key proteins (and lipids) in microvilli remains unknown. Future works are required to discover these important organizers of signalling receptors at the plasma membrane of T cells. Such research may open new avenues for treatment of many human diseases, which are associated with the malfunction of these critical immune cells.

## Author Contributions

The author confirms being the sole contributor of this work and has approved it for publication.

## Funding

This work was supported by Czech Science Foundation (19-07043S).

## Conflict of Interest

The author declares that the research was conducted in the absence of any commercial or financial relationships that could be construed as a potential conflict of interest.
